# Bleeding versus Clotting: A Complex Case of a Large Fibroid Uterus Causing Menorrhagia and a DVT

**DOI:** 10.1155/2016/4169565

**Published:** 2016-08-14

**Authors:** Sangeeta Ramanan, Jude Chapman-Wardy, Roy Watson

**Affiliations:** ^1^Lyell McEwin Hospital, Adelaide, SA 5112, Australia; ^2^Modbury Hospital, Adelaide, SA 5092, Australia; ^3^The Queen Elizabeth Hospital, Adelaide, SA 5011, Australia

## Abstract

A 43-year-old woman presented with severe anaemia secondary to menorrhagia. Pelvic ultrasound showed a large intramural posterior fundal fibroid. Hysteroscopy showed the fibroid distorting the endometrial cavity, precluding Mirena® device insertion. As she was initially hesitant to have a hysterectomy, medical management with the oral contraceptive pill (OCP) and tranexamic acid was instituted, with good effect. Months later, after a long road trip, she presented with left leg swelling, and a Doppler ultrasound confirmed an extensive deep vein thrombosis (DVT). She was commenced on warfarin for anticoagulation but presented again with menorrhagia precipitated by overanticoagulation. After initial stabilization with multiple blood transfusions and reversal of anticoagulation, the warfarin was ceased in favour of enoxaparin and she underwent inferior vena cava (IVC) filter insertion prior to a total abdominal hysterectomy. Mass effect from large uterine fibroids can cause venous thromboembolism (VTE). A duplex ultrasound of the lower limbs if a woman presents with a large fibroid could identify asymptomatic DVTs in such women. A prehysterectomy IVC filter would then reduce their risk of postoperative pulmonary embolism. Medical management of menorrhagia with procoagulants should be avoided for management of menorrhagia in such women given their higher risk of developing VTE.

## 1. Introduction

Virchow's triad of venous stasis, endothelial damage, and hypercoagulability has been used to describe the pathogenesis of venous thromboembolism for over a century [1].

Venous stasis is caused by immobility due to recent surgery or illness or a sedentary lifestyle. Stasis is also due to any obstruction to blood flow. Endothelial damage results from trauma, such as lower limb fractures, or from excess venous compression during surgery or travel. A hypercoagulable state is caused by multiple factors, such as malignancy, pregnancy, polycythaemia, thrombocytosis, acquired and inherited thrombophilias, use of exogenous oestrogen (e.g., the oral contraceptive pill or hormone replacement therapy) [2], and other medications such as tranexamic acid [3].

Leiomyomas or uterine fibroids are benign, monoclonal tumours of the smooth muscle cells in the uterus [4]. Women with fibroids can remain asymptomatic [5] or may have symptoms of abnormal uterine bleeding (menorrhagia, prolonged bleeding, etc.), dyspareunia, and pelvic pain [6].

Women with leiomyomas are also at an increased risk of developing venous thromboembolism due to multiple different mechanisms. Polycythaemia and reactive thrombocytosis have been seen in women with menorrhagia due to fibroids [2], and these are risk factors for venous thromboembolism [7, 8]. Mass effect from benign space occupying lesions, including large uterine fibroids, can result in venous stasis of the lower limbs, leading to venous thromboembolism (VTE) [2, 9–12]. Pathological, nonuniform enlargement of the uterus is more likely to cause impingement of pelvic veins than uniform enlargement of the uterus (as seen in pregnancy) [2].

We report a case of a multiple risk factors leading to a massive deep venous thrombosis in a woman with a large uterine leiomyoma.

## 2. Case Presentation

A 43-year-old woman presented with severe anaemia secondary to menorrhagia. She was symptomatic of dizziness, shortness of breath on exertion, and worsening fatigue. She reported having increasingly heavy and painful periods for 2 years, requiring 5-6 pads per day. She had regular 30-day cycles, and her periods lasted 5-6 days. She had previously tried oral contraceptive pills (OCP) but had experienced headaches with their use and hence had ceased them. She was a nonsmoker and had had one vaginal delivery 16 years ago.

On examination, she was found to be tachycardic but normotensive. She had a soft nontender abdomen. Her uterus was enlarged and palpable and corresponded to 16 weeks' gestation. On vaginal examination, there was a mass felt in the pouch of Douglas. Her cervix appeared normal.

Her haemoglobin was 55 g/L. Pelvic ultrasound showed an intramural posterior fundal fibroid measuring 9.3 × 9.1 × 10.8 cm. Endometrial thickness was 3 mm.

After initial resuscitation with IV fluids and 3 units of packed red blood cells, she underwent a hysteroscopy, which showed the fibroid distorting the endometrial cavity, precluding endometrial biopsy and Mirena® device placement. As she was initially hesitant to have a hysterectomy, medical management was instituted with tranexamic acid and a different OCP than that used previously, with good effect.

Fifteen months later, she presented again on day 2 of her periods after a syncopal episode at home. She had a postural drop in her blood pressure (99 mmHg to 76 mmHg systolic from lying to standing). She was admitted for observation and given tranexamic acid to stop her bleeding. Her haemoglobin dropped from 89 g/L to 65 g/L during that admission. She was again counselled regarding permanent methods of management of menorrhagia and consented to a hysterectomy.

While awaiting her hysterectomy, she went on a long road trip and presented again with left leg swelling and tightness. She denied chest pain or shortness of breath. Circumferential measurements of her legs supported the diagnosis of a deep vein thrombosis (DVT) (Table 1).

**Figure 1:**
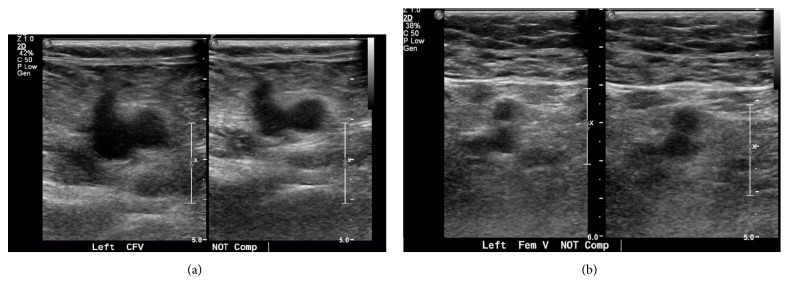
Doppler US of left lower limb showing DVT in the left common femoral (a) and femoral veins (b).

Doppler ultrasound of the left limb showed an extensive deep vein thrombosis involving the iliac, femoral, popliteal, posterior tibial, and gastrocnemius veins (Figure 1). The uterine fibroid was found to be compressing the left common iliac vein.

**Figure 2:**
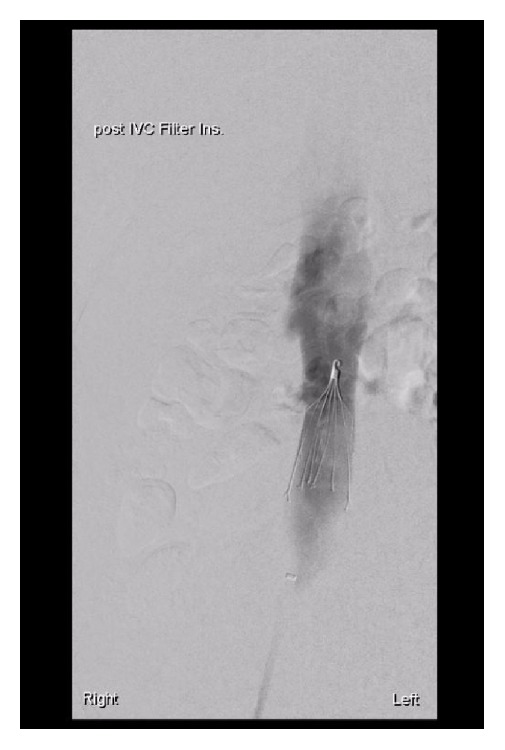
Venogram showing IVC filter.

She was commenced on warfarin for anticoagulation but presented again with menorrhagia and was found to be severely anaemic and overanticoagulated. Her haemoglobin was 54 g/L and INR 6.6. She was stabilized with multiple blood transfusions and reversal of anticoagulation. After consultation with a haematologist, her warfarin was ceased in favour of enoxaparin. She underwent fluoroscopy-guided inferior vena cava (IVC) filter insertion (Figure 2) prior to a total abdominal hysterectomy and bilateral salpingectomy. Significant endometriosis was an unexpected incidental intraoperative finding.

**Figure 3:**
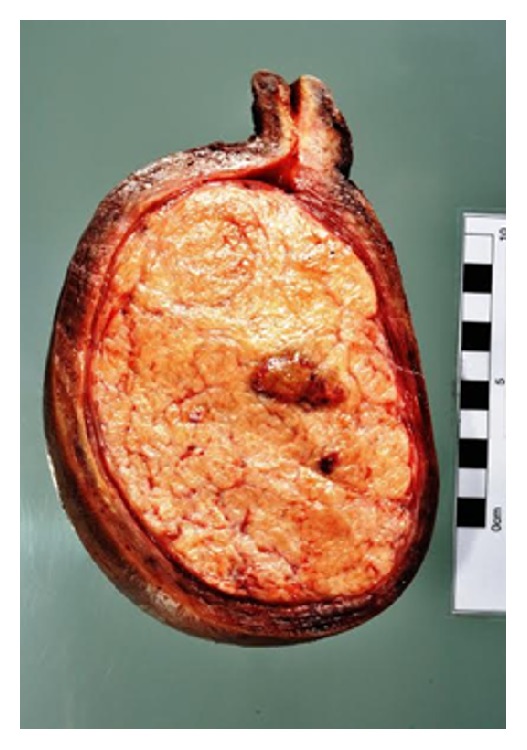
Cross section of uterus with large leiomyoma.

Histology confirmed a benign leiomyoma measuring 14 × 9 cm with foci of red degeneration and minor adenomyosis (Figure 3). The uterus weighed 1490 g.

The patient was restarted on enoxaparin 6 hours postoperatively and underwent removal of the IVC filter a few weeks after the hysterectomy.

## 3. Discussion

In this case, the patient had multiple factors contributing to the development of her DVT. The large fibroid uterus compressed her left common iliac vein, causing venous stasis. She also had been on a long road trip, and this period of prolonged immobility resulted in further venous stasis. Her severe menorrhagia was treated with the oral contraceptive pill and tranexamic acid, both of which have a procoagulant effect.

Women with large uterine fibroids may have asymptomatic DVTs that they and their clinicians may be unaware of. A hysterectomy to treat symptoms caused by the fibroid(s) could potentially release the occlusive effect of the fibroid uterus on deep pelvic veins. This may inadvertently lead to a pulmonary embolus immediately after hysterectomy, which can be fatal.

Duplex ultrasound of the lower limbs should be considered if a woman presents with a large fibroid uterus. This could identify asymptomatic DVTs in such women. A prehysterectomy IVC filter would then reduce their risk of postoperative pulmonary embolism.

Large fibroids usually result in menorrhagia. In many instances, this is managed medically with the oral contraceptive pill and/or tranexamic acid, while awaiting definitive surgery. However, the procoagulant effect of these drugs increases the risk of venous thromboembolism in such women [13, 14].

Medical management of menorrhagia with procoagulants (e.g., the oral contraceptive pill or tranexamic acid) should be avoided for management of menorrhagia in such women given their higher risk of developing VTE. Thromboprophylaxis must be offered to women with large leiomyomas who plan to undertake long trips [15].

## Figures and Tables

**Table 1:** Calf and thigh circumferences of the patient's lower limbs.

	Left	Right
Calf circumference (cm)	37.5	34
Thigh circumference (cm)	51.5	43.5

## References

[1] Kumar D. R., Hanlin E. R., Glurich I., Mazza J. J., Yale S. H. (2010). Virchow's contribution to the understanding of thrombosis and cellular biology. *Clinical Medicine and Research*.

[2] Fletcher H., Wharfe G., Williams N. P., Gordon-Strachan G., Pedican M., Brooks A. (2009). Venous thromboembolism as a complication of uterine fibroids: a retrospective descriptive study. *Journal of Obstetrics and Gynaecology*.

[3] Mannucci P. M., Levi M. (2007). Prevention and treatment of major blood loss. *The New England Journal of Medicine*.

[4] Parker W. H. (2007). Etiology, symptomatology, and diagnosis of uterine myomas. *Fertility and Sterility*.

[5] Okolo S. (2008). Incidence, aetiology and epidemiology of uterine fibroids. *Best Practice and Research: Clinical Obstetrics and Gynaecology*.

[6] Zimmermann A., Bernuit D., Gerlinger C., Schaefers M., Geppert K. (2012). Prevalence, symptoms and management of uterine fibroids: an international internet-based survey of 21,746 women. *BMC Women's Health*.

[7] Akins P. T., Glenn S., Nemeth P. M., Derdeyn C. P. (1996). Carotid artery thrombus associated with severe iron-deficiency anemia and thrombocytosis. *Stroke*.

[8] Voigt W., Jordan K., Sippel C., Amoury M., Schmoll H.-J., Wolf H. H. (2008). Severe thrombocytosis and anemia associated with celiac disease in a young female patient: a case report. *Journal of Medical Case Reports*.

[9] Shiota M., Kotani Y., Umemoto M. (2011). Deep-vein thrombosis is associated with large uterine fibroids. *The Tohoku Journal of Experimental Medicine*.

[10] Rosenfeld H., Byard R. W. (2012). Lower extremity deep venous thrombosis with fatal pulmonary thromboembolism caused by benign pelvic space-occupying lesions—an overview. *Journal of Forensic Sciences*.

[11] Nishikawa H., Ideishi M., Nishimura T. (2000). Deep venous thrombosis and pulmonary thromboembolism associated with a huge uterine myoma: a case report. *Angiology*.

[12] Riat R., Chowdary P., Mavrides E., Magos A., Gatt A. (2013). Is there an Association between Thrombosis and Fibroids? A single centre experience and literature review. *International Journal of Laboratory Hematology*.

[13] Vandenbroucke J. P., Rosing J., Bloemenkamp K. W. M. (2001). Oral contraceptives and the risk of venous thrombosis. *The New England Journal of Medicine*.

[14] Sundström A., Seaman H., Kieler H., Alfredsson L. (2009). The risk of venous thromboembolism associated with the use of tranexamic acid and other drugs used to treat menorrhagia: a case-control study using the General Practice Research Database. *BJOG: An International Journal of Obstetrics and Gynaecology*.

[15] Philbrick J. T., Shumate R., Siadaty M. S., Becker D. M. (2007). Air travel and venous thromboembolism: a systematic review. *Journal of General Internal Medicine*.

